# Self-rated exhaustion disorder and associated health-related factors among municipal employees in rural areas of northern Sweden

**DOI:** 10.1007/s00420-020-01617-3

**Published:** 2020-12-09

**Authors:** Sofia Asplund, Johan Åhlin, Sture Åström, Mattias Hedlund, Britt-Marie Lindgren, Eva Ericson-Lidman

**Affiliations:** 1 Department of Nursing, Umeå University, 90187 Umeå, Sweden; 2 Department of Community Medicine and Rehabilitation, Umeå University, Umeå, Sweden

**Keywords:** Self-rated exhaustion disorder, Health, Work-related stress, Municipal employees, Rural areas

## Abstract

**Objective:**

The aims of this study among municipal employees in rural areas of northern Sweden were to assess the prevalence of self-rated exhaustion disorder (s-ED), describe plausible between-group differences in self-reported health-related factors among employees with or without s-ED, and identify health-related factors associated with s-ED.

**Methods:**

In a cross-sectional study, data were collected from 1093 municipal employees (76.1% women) in two rural areas using an instrument measuring s-ED and health variables drawn from the Modern Worklife Questionnaire (MWQ), the Perceived Stress Scale (PSS), and the National Board of Health and Welfare’s questions about physical activity. Comparisons were made between an s-ED and a non-s-ED group. Health-related factors associated with s-ED were identified through a logistic regression.

**Results:**

Self-rated exhaustion disorder was reported by 21.5% of the participants. Health-related factors associated with s-ED were cognitive problems, sleep problems, depressive symptoms, high stress, poor self-rated health, and stomach problems. There was no statistically significant difference in the prevalence of participants who met the criteria of physical activity among s-ED and non-s-ED group.

**Conclusion:**

Findings from this study suggest that s-ED is more common among municipal employees in rural areas than in other working populations in Sweden. Several health-related factors were associated with s-ED. Regular use of a self-rated instrument in evaluating the organizational and social work environment can identify people at risk of developing exhaustion disorder and requiring long-term sick leave.

## Introduction

In Sweden, as in many other countries, there is an increasing trend of mental ill health. Stress-related disorders, mainly due to psychosocial stress are causing about half of the long-term sick leave in the country. Among the stress-related disorders, almost every fifth sick leave case among women is caused by stress-related exhaustion, and the sick leave often lasts over six months. A large part of the long-term sick leave can be linked to our working life, and an imbalance between work and private life (Swedish Social Insurance Agency 2020). Organizational and social issues, such as conflicts and high demands at work, can lead to harmful psychosocial workload and long-term sick leave (Holmgren et al. 2009; Holmgren, Fjällström-Lundgren, & Hensing 2013). Occupations in human service sectors, such as health care, social care, and education, are at high risk for sick leave due to stress-related disorders (Health and Safety Executive 2018). The highest rates of sick leave in Sweden occur among employees in the municipal sector. Stress-related disorders dominate, and women’s sick leave rates are twice as high as men’s. Municipal employees in rural areas of northern Sweden have among the highest rates of long-term sick leave in the country (Swedish Association of Local Authorities and Regions 2019). Psychosocial stress exposure among municipal employees, with its accompanying risk of exhaustion disorder (ED), is thus a natural focus of this study.

ED is a medical diagnosis formulated in Sweden to facilitate the classification of patients seeking care for stress (ICD-10 code F43.8A; National Board of Health and Welfare 2020). To fulfil the diagnostic criteria, a person must have been exposed to stress for 6 months or more. ED is characterized by lack of energy and pronounced physical and mental exhaustion. Mental and physical symptoms include sleeping disorders, problems with concentration and memory, gastrointestinal symptoms, and headache. Stress can arise from work, private life, or a combination of both (National Board of Health and Welfare 2003). Hasselberg et al. (2014) found among patients with ED that high work demands are the greatest perceived stressor, followed by private relationship problems, and it is often impossible to distinguish between stress from working and private life. The term “self-rated exhaustion disorder” (s-ED) is based on the Swedish diagnostic criteria for ED, but it also includes individuals’ own perceptions of the severity of their condition and its effect on their well-being and functional capacity (Glise et al. 2010). It is important to study the work environment, as it is associated with developing ED.

The work environment is an important factor in employee health. One definition of health from the perspective of promoting health in workplaces is “feeling good and having sufficient resources to meet everyday demands and being able to realize both personal and professional goals” (Hultberg et al. 2018). Many aspects of our lifestyle and work conditions can determine how we experience health, and most of Sweden’s population (80%) perceive that they are in good health (Mood 2013; Slavich 2016). Many health-related factors (e.g., sleep disturbance, anxiety, headache, gastrointestinal symptoms, and physical inactivity) are associated with ED (Adamsson and Bernhardsson 2018; Wallensten et al. 2019). Physical activity has found to be associated with resilience to stress-related disorders (Jonsdottir et al. 2010), as well as effective in the rehabilitation of people suffering from ED (Gerber et al. 2015). The association between health-related factors and s-ED among municipal employees in rural areas of Sweden has not previously been studied.

International research has stated that living in a rural area can be a risk factor of health (Anderson et al. 2015; Befort et al. 2012; McLaughlin et al. 2013; Trivedi et al. 2015). This is also true in Sweden, where rural populations in northern Sweden have higher incidences of cardiovascular disease, obesity, and physical inactivity (Klingberg et al. 2019; Lindroth et al. 2014). The Swedish Board of Agriculture (2015) divides Sweden’s municipalities into different categories based on population density and proximity to a city. In rural and sparsely populated rural areas, most or all of the population live in rural areas, have more than 45 min away from a city of at least 50,000 inhabitants. The term “rural areas” is used throughout the article.

Little is known regarding s-ED and health-related factor associations among municipal employees in rural areas of northern Sweden, and the area of interest has rarely been focus in previous research studies. This study will focus on s-ED among this working population.

The aims of this study among municipal employees in rural areas of northern Sweden were to assess the prevalence of self-rated exhaustion disorder (s-ED), describe plausible between-group differences in self-reported health-related factors among employees with or without s-ED, and identify health-related factors associated with s-ED.

## Methods

### Study design

In a cross-sectional study, data were collected through a web-based questionnaire from March to June 2018. A link to the questionnaire was sent by email to the employees and a paper-based questionnaire was sent to those few without an email address. Three reminders were sent to non-responders. The questionnaire collected both background variables (e.g., age, sex, work, and education) and responses to instruments measuring s-ED, stress, health, and physical activity. The study was planned with the municipal authorities in two rural municipalities in northern Sweden.

### Settings and subjects

The study was limited to two rural municipalities in northern Sweden. Municipality 1 has about 3100 inhabitants in an area of 1600 square kilometres (~ 618 square miles) and Municipality 2 has about 12 200 in 5500 square kilometres (~ 2125 square miles; SCB 2018). The participants were all municipal employees. A total of 2077 employees were asked to participate in the study, 1093 (52.6%) of whom completed the questionnaire. Three people were excluded in the summery of the s-ED scale because of missing internal values, which made it impossible to categorize them as s-ED or non-ED. In all, 1090 people completed the s-ED scale. The characteristics of the participants are summarized in Table 1.

**Table 1:** Characteristics of the participants

	Municipal 1 *n* = 267 (24.4%)	Municipal 2 *n* = 826 (75.6%)	Total *n* = 1093 (100%)	*p* value	Effect size
Sex					
Male (%)	58 (21.7)	203 (24.6)	261 (23.9)	0.385	*φ* = − 0.029
Female (%)	209 (78.3)	623 (75.4)	832 (76.1)		
Age (range 19–67)					
Mean years ± SD	45.3 ± 11.8	45.0 ± 12.1	45.1 ± 12.0	0.741	d = 0.023
Employment					
Work full time (%)	196 (73.4)	591 (71.7)	787 (72.1)	0.649	φ = 0.016
Work part time (%)	71 (26.6)	233 (28.3)	304 (27.9)		
Time at current workplace (range 0–42)					
Mean years ± SD	9.0 ± 8.6	8.6 ± 8.7	8.7 ± 8.7	0.548	d = 0.043
Time as municipal employee (range 0–45)					
Mean years ± SD	13.9 ± 10.6	15.7 ± 11.2	15.2 ± 11.1	0.022	d = 0.165
Long-term sick leave					
No (%)	247 (92.5)	780 (94.4)	1027 (94.0)	0.318	φ = − 0.035
Yes (%)	20 (7.5)	46 (5.6)	66 (6.0)		
Children living at home					
Yes (%)	136 (51.7)	389 (47.1)	525 (48.2)	0.217	φ = − 0.040
No (%)	127 (48.3)	437 (52.9)	564 (51.8)		
Working schedule					
Day/evening (%)	203 (76.0)	677 (82.0)	880 (80.5)	0.042	φ = − 0.064
Night/Shift (%)	64 (24.0)	149 (18.0)	213 (19.5)		
Marital status					
Living with a partner (%)	218 (81.6)	643 (77.8)	861 (78.8)	0.393	V = 0.041
Living apart together (%)	8 (3.0)	26 (3.2)	34 (3.1)		
Single (%)	41 (15.4)	157 (19.0)	198 (18.1)		
Education					
Compulsory school (%)	12 (4.5)	29 (3.5)	41 (3.8)	0.637	V = 0.029
Upper secondary school (%)	125 (46.8)	408 (49.4)	533 (48.7)		
University (%)	130 (48.7)	389 (47.1)	519 (47.5)		
Living					
Urban area (%)	172 (64.4)	637 (77.1)	809 (74.0)	< 0.001	φ = − 0.124
Rural area (%)	95 (35.6)	189 (22.9)	284 (26.0)		
Home					
House (%)	212 (79.7)	559 (67.7)	771 (70.6)	0.001	V = 0.114
Apartment (%)	53 (19.9)	260 (31.5)	313 (28.7)		
Other (%)	1 (0.4)	7 (0.8)	8 (0.7)		
Profession					
Nursing staff (%)	94 (35.2)	283 (34.3)	377 (34.6)	0.002	V = 0.126
Educational staff (%)	90 (33.7)	366 (44.4)	456 (41.8)		
Managers (%)	19 (7.1)	58 (7.1)	77 (7.1)		
White-collar workers (%)	42 (15.7)	76 (9.2)	118 (10.8)		
Blue and pink collar workers (%)	22 (8.2)	41 (5.0)	63 (5.8)		

### Instruments

#### Self-rated exhaustion disorder

A self-report instrument was used to determine whether people met the criteria for s-ED. Such self-reported criteria do not indicate a medical diagnosis of ED, but they can be used preventatively at workplaces to catch early signs of exhaustion. Self-rated exhaustion was measured on the Self-rated Exhaustion Disorder (s-ED) scale, fully described by Glise et al. (2010). The instrument consists of nine questions based on the diagnostic criteria for ED, for example: “Do you currently feel, and have felt for more than 2 weeks, physically and/or mentally exhausted?” The answer options are yes or no. To be classified into the s-ED group, participants must have answered yes to questions 1, 2, and 4 and have met at least four of the six conditions in question 3. The instrument distinguishes between light/moderate and pronounced s-ED and has been validated in a study of health in medical staff, which showed sufficient construct validity. The s-ED scale is considered very suitable for identifying employees with stress-related mental health problems at an early stage (Glise et al. 2010).

#### Self-rated health

Health variables were measured using 17 questions from the Modern Worklife Questionnaire (MWQ). The questions are divided into eight categories: sleep, physical symptoms, headache, stomach problems, cognitive problems, depressive symptoms, self-rated health, and work ability. The mean score for each category is based on one or several questions, such as “How do you sleep?” and “How do you rate your general state of health?” Questions have between two and seven response options ranging from very good to very bad. The MWQ contains 127 questions focused on organizational and social work environment to (1) examine causes of stress in an organization and (2) measure factors in work life related to ill health (e.g., demands, control, and support). The results from a workplace can be compared with the average for the working Swedish population in the national Swedish Longitudinal Occupational Survey of Health database. Cronbach’s alpha for health-related factors was found to be between 0.59 and 0.89 (Oxenstierna et al. 2008a Oxenstierna et al. 2008b).

#### Perceived stress

The Perceived Stress Scale (PSS) is used to measure people’s perception of stress (Cohen et al. 1983). The original instrument was a 14-item scale (PSS-14), but it has been shortened to 10 items (PSS-10). The purpose of the instrument is to assess how much stress a person experienced in the last month. The respondent estimates how often life seemed unpredictable, uncontrollable, and/or overloaded. Each of the items on the PSS-10 are rated on a 5-point Likert scale ranging from 0 (never) to 4 (very often). The PSS-10 consists of six positive and four negative items. The total score on the scale ranges from 0 to 40 (higher scores representing higher levels of stress; Cohen et al. 1983). A mean score of ≥ 20 is categorized as “high stress” and < 20 as “low stress” (Schwarz et al. 2016). The 10 items included in the PSS-10 were translated by Eskin and Parr (1996), and the Swedish translation has shown good validity (Nordin and Nordin 2013). Cronbach’s alpha for the PSS-10 was between 0.74 and 0.91 in 12 studies (Lee 2012).

#### Physical activity

Physical activity was measured on the National Board of Health and Welfare's three-question questionnaire. Question 1 measures time in physical training: “During a regular week, how much time do you spend exercising at an intensity that makes you breathless, for example, running, fitness class, or ball games?” Six response options range from 0 to more than 120 min. Question 2 measures non-exercise physical activity: “During a regular week, how much time are you physically active in ways that are not exercise, for example, walking, bicycling, or gardening? Add together, all activities lasting at least 10 min.” Seven response options range from 0 to more than 300 min. Question 3 measures sedentary activity. The high-intensity and low-intensity physical activity scores are summed and a total score of ≥ 11 is considered to meet the recommended level of activity. The questions have been validated (Kallings and Börjesson 2014).

### Ethics

The study was approved by the Regional Ethical Review Board in Umeå, Sweden (Dnr 2017/495–31). In an introductory letter, the employees were carefully informed about the voluntary nature of participating before being emailed the link to the questionnaire. A completed questionnaire was considered consent to participate in the study and results were presented on the group level. Throughout the study, the participants’ human rights were protected, and the study was conducted according to the 2013 Declaration of Helsinki (WMA 2013).

### Statistical analysis

The Statistical Package for Social Sciences version 25.0 was used for all calculations (IBM 2017). An “s-ED” variable was created based on the s-ED scale, grouping the municipal employees into s-ED (light/moderate/pronounced s-ED) or non–s-ED. Descriptive statistics are presented as mean scores, standard deviations, and frequency distributions when applicable. Group comparisons were made using *t* test and chi-square depending on the characteristics of the variable. The effect sizes used in addition to statistical significance were analysed using Cohen’s *d* (*d*), phi coefficient (*Φ*), and Cramer’s *V* (*V*). Cohen’s criteria define values above 0.2 as small, above 0.5 as medium, and above 0.8 as large effects (Cohen 1988, Ch. 2). Phi coefficient values above 0.10 represent small effects, above 0.30, moderate, and above 0.5, large (Cohen 1988, ch.7). The criteria for Cramer’s V vary depending on the number of categories; we used the criteria described by Pallant (2016, p. 221). A logistic regression analysis was used to identify health-related factors independently associated with s-ED and to estimate odds ratios (ORs) and 95% confidence intervals (CIs). Variables from Table 2 with at least a medium effect size and a *p* value of < 0.05 were put through logistic regression analysis. Variables associated with s-ED were tested for multicollinearity using Pearson’s *r*; *r* > 0.7 was considered an indication of multicollinearity, as recommended by Pallant (2016, p. 159). A variance inflation factor of > 10 was also considered an indication of multicollinearity, as was a tolerance value of > 0.1, also suggested by Pallant (2016, p. 159). A *p* value < 0.05 was considered to indicate statistical significance in all calculations. The ordinal variables were dichotomized (e.g., stomach problems [yes, often; yes, sometimes] vs. no stomach problems [no, rarely; no, never]). A logistic regression analysis (Table 3) was performed with s-ED as the dependent factor, and high stress, sleep problems, stomach problems, depressive symptoms, cognitive problems, and poor self-rated health as independent factors (categorical) as they met the criteria (described above) for inclusion in the model.

**Table 2:** Health comparisons between municipal employees with and without s-ED (n = 1090)

	s-ED *n* = 234	non s-ED *n* = 856	*p* value	Effect size
Women (%)	199 (85)	632 (75)	< 0.001	*φ* = 0.108
Age ± SD	42.35 ± 12.32	45.80 ± 11.88	< 0.001	*φ* = 0.285
Not physically active (%)	112 (48)	471 (55)	0.061	*φ* = 0.059
High stress (%)	153 (65)	108 (13)	< 0.001	*φ* = 0.508
Sleep problems (%)	188 (80)	268 (31)	< 0.001	*φ* = 0.408
Physical symptoms^a^ (%)	216 (92)	628 (73)	< 0.001	*φ* = 0.186
Headache (%)	197 (84)	463 (54)	< 0.001	*φ* = − 0.253
Stomach problems^b^ (%)	173 (74)	303 (35)	< 0.001	*φ* = 0.319
Depressive symptoms^c^ (%)	160 (68)	97 (11)	< 0.001	*φ* = 0.552
Cognitive problems^d^ (%)	184 (79)	180 (21)	< 0.001	*φ* = 0.502
Poor work ability (%)	83 (36)	90 (11)	< 0.001	*φ* = 0.280
Poor self-rated health (%)	168 (72)	183 (21)	< 0.001	*φ* = 0.443

^a^Pain in back, neck, shoulders, arms or legs

^b^Heartburn, acid reflux, abdominal pain, or upset stomach

^c^Lack of energy, depression, anxiety, lack of interest, self-blame, and exhaustion

^d^Concentration problems, difficulty making decisions, memory problems, and difficulty thinking

**Table 3:** Logistic regression analysis of health-related factors associated with s-ED

	Wald	OR/Exp	95% CI
Cognitive problems	42.57	4.22	2.74–6.51
Sleep problems	32.66	3.57	2.31–5.53
Depressive symptoms	24.71	3.18	2.02–5.02
High stress	18.20	2.67	1.70–4.18
Poor self-rated health	19.91	2.60	1.71–3.95
Stomach problems	15.73	2.34	1.54–3.56

Model *χ*: 513.991 (*df* 6, *p* value < 0.001). The model explained between 37.6% (Cox and Snell R Square) and 58.1% (Nagelkerke *R* square)

## Results

Table 1 presents the background characteristics of the municipal employees in two rural areas of northern Sweden. Of the 1093 participants, 267 (24.4%) lived in Municipality 1 and 826 (75.6%) in Municipality 2. There were 832 (76.1%) women and 261 (23.9%) men. The mean age was 45.1 years and mean time as a municipal employee, 15.2 years. Close to half (48.2%) had children at home and most worked day or evening shifts (80.5%). In addition, most worked in education (41.8%) or nursing (34.6%). Nearly half (47.5%) had a university education.

Table 2 shows comparisons between municipal employees with and without s-ED. Among the 1090 who completed the s-ED scale, 234 (21.5%) reported some level of s-ED. Within the group of participants with s-ED, there was a significantly higher proportion of women (85%) then men (15%; *p* < 0.001). Of those with s-ED, a significantly higher proportion experienced high stress (65% vs. 35% with low stress; *p* < 0.001), depressive symptoms (68% vs. 32% with no depressive symptoms; *p* < 0.001), and cognitive problems (79% vs. 21% with no cognitive problems; *p* < 0.001). There was no significant between-group difference in the proportions of participants who met or did not meet the physical activity recommendations (Table 2).

The logistic regression analysis (Table 3) showed that cognitive problems (OR 4.22) was the strongest factor predicting s-ED. This means that the employees with cognitive problems were more than four times more likely to report s-ED than those without. Participants with sleep problems were 3.57 times likely to report s-ED than those with good sleep, and those with depressive symptoms more than three times more likely (OR 3.18) to report s-ED than those without. Municipal employees with high stress were 2.67 times more likely to report s-ED than those with low stress, and those with poor self-rated health were two and a half (OR 2.60) times more likely to report s-ED than those with good self-rated health. Stomach problems (OR 2.34) were the weakest of the six predicting factors, but those who experienced stomach problems were still over two times likely to report s-ED than those without. The model explained between 37.6% and 58.1% of the variance of s-ED.

## Discussion

In this study, the prevalence of s-ED and associated health-related factors were described among municipal employees in two rural municipalities in northern Sweden. We found that s-ED was relatively common among the employees, and that cognitive problems, sleep problems, depressive symptoms, high stress, poor self-rated health, and stomach problems were the health-related factors most strongly associated with s-ED. No difference was found regarding employees who met the physic activity recommendations among those with or without s-ED.

The results showed that 21.5% of the municipal employees reported having s-ED. Other Swedish studies using the same instrument found an s-ED prevalence rate of 7.8–19% among county council healthcare workers, social insurance officers, and the general population in southern Sweden (Ahlborg et al. 2016; Glise et al. 2010; Persson et al. 2016). No previous studies have investigated the prevalence of s-ED exclusively among municipal employees in rural areas; it is, therefore, difficult to make comparisons. From this study, it is not possible to draw conclusions about whether or how much the prevalence rate may be attributed to municipal employment and/or to living in a rural area. Similar prevalence rates, however, were found in the previously described studies conducted in human service occupations.

The highest sick leave rate in Sweden is found within the municipal sector (Swedish Association of Local Authorities and Regions 2019), which might suggest employment as the primary driver of s-ED. However, populations in many of Sweden’s rural municipalities are decreasing, resulting in consequences, such as school closures, centralization of elderly care, staff cuts, and increased income tax. Employees of municipalities with decreasing populations have higher rates of long-term sick leave than those in other Swedish municipalities, and rural municipalities can also have difficulty recruiting skilled employees (Syssner et al. 2017). How the employer organizes the work place and schedules duties is also important to employee health and sick leave rates. Promoting health in workplaces and improving both leadership styles and the social climate are important in lowering employee exhaustion levels (Åkerlind et al. 2013), and a stress management intervention has shown positive effects in Sweden (Grossi and Santell 2009). More comparative research is needed to decide whether and to what extent municipal employment and/or the rural context is related to s-ED. However, both preventive interventions and the efforts taken to improve the organizational and social work environments in the two rural municipalities in this study may help employees to stay healthy in their workplace.

The results showed that of several health-related factors associated with s-ED, cognitive problems and sleep problems were the two strongest. This is in line with other research showing that cognitive problems were the most pronounced symptom reported by patients with ED, and attention/working memory could remain impaired several years (Ellbin et al. 2018; Jonsdottir et al. 2017, 2013). Previous research has also related sleep problems to ED, describing disturbed sleep as the best predictor of ED (Adamsson and Bernhardsson 2018; Grossi et al. 2015). A review found that sleep disruption is not only a predictor of ED, but also contributes to the onset and possibly the continuance of ED (Wallensten et al. 2019).

Other health-related factors associated with s-ED in the present study were depressive symptoms, high stress, poor self-rated health, and stomach problems. Other research found that depressive symptom preceding ED was the most commonly symptom reported by school staff, and was experienced by half of the participants (Adamsson and Bernhardsson 2018). A review of prospective studies among working populations reported job burnout to predict health problems, such as depressive symptoms (Salvagioni et al. 2017). In another working population study aged 18–65 years, depressive symptoms were common among those who reported high stress. Among those with high stress, ED was present in 74% of the participants. Among those with lower stress, ED was present among 32% of the participants (Wiegner et al. 2015). In a study by Adamsson and Bernhardsson (2018), stress was the most common symptom in healthcare workers diagnosed with ED. Poor self-rated health has found to be a useful marker for increased risk of work disability, and also associated with increased sick days among municipal employees (Pietilainen et al. 2011; Voss et al. 2008), as well as long-term sick leave in health care professionals (Peterson et al. 2011). Somatic symptoms have been reported by almost all (98%) in a study with 234 participants with ED, and the most common symptom was stomach problems (reported by 67%). The number of symptoms reported was significantly related to the severity of mental health problems (Glise et al. 2014). This implies that it is important to be aware of several health symptoms that could indicate signs of stress, and part of developing ED among municipal employees.

In this study, the prevalence of participants meeting physical activity recommendations among those with or without s-ED did not differ. This result was somewhat surprising. Wallensten et al. (2019) reported that in patients with ED, physical activity had a positive effect on reducing depressive symptoms and also lowered levels of burnout. A recent review showed that physical activity interventions appear to be effective in coping with stress and improving mental health (Sharon David and Tenenbaum 2017). Self-reported physical exercise has also been shown to play a role in discriminating between burnout and non-burnout groups (Peterson et al. 2008). The finding that the degree of physical activity was not associated with ED must be interpreted with some caution. A single measurement of self-rated physical activity does not distinguish between the realms of leisure, work, and commuting (Holtermann et al. 2018) and is probably not sufficient to conclude that physical activity and training are not associated with ED. Long-term follow-up studies are needed to explore the plausible effects of regular physical activity and physical training in this context.

### Methodological considerations

Some strengths and limitations of this study must be addressed. A strength was that all municipal employees in the two municipalities were invited to participate, and a relatively large sample answered, with only a few internal missing values among the respondents. The study had a response rate of 52.6%, which is somewhat higher that could be expected from web-based studies (Blumenberg and Barros 2018). Our sample can be considered representative due to sex and age when compared to national data (Swedish Association of Local Authorities and Regions 2018). Still, some caution should be taken when interpreting and generalizing the results. One limitation is the cross-sectional design, which undermines any deductions about causal relationships between the study variables. This study was based on self-reports of exhaustion, there were no objective measurements with assessments from professionals from health care. In this study, self-reports of the method used made it possible to identifying individuals who could be at risk of developing ED and long-term sick leave. In studies with self-reports, subjects could exaggerate or underreport health problems, which could lead to interpretation difficulties. However, self-reports assessments have an important role in stress research as the first step in identifying risk groups, and risk exposures (Theorell and Hasselhorn 2005). Self-reported measures of physical activity have been demonstrated to have weak agreement with objective measurements (Steene Johannessen et al. 2016). This study was limited to two municipalities, and is therefore not a representative sample of the Swedish overall population. However, it is reasonable to believe that these results could be transferred to other rural areas in Sweden, since many other rural municipalities in Sweden have, e.g. similar organization structure, problems with depopulation, economy, and long-term sick leave. Since ED is not used as a medical diagnosis in other countries, and the s-ED scale is based on the diagnosis, caution must be taken extrapolating our findings to other countries.

## Conclusion

More than every fifth municipal employees in rural areas of northern Sweden reported s-ED, even though they were not on sick leave. The prevalence rate in this study was somewhat higher than other working populations in Sweden. Several physical and psychological health-related factors were associated with s-ED. This means that workplace awareness is important, since health impairment could indicate a development of ED. For the employer, screening workplaces with questionnaires, such as the s-ED scale, could be part of the organizational work. Organizational preventive interventions could be one way to prevent ED, and long-term sick leave. Future research should focus on gaining more knowledge and understanding of s-ED in rural areas through, e.g. longitudinal studies. An upcoming study will focus on factors in the organizational and social work environment, and the associations with s-ED.

## Data Availability

The data that support the findings of this study are available on request from the corresponding author.

## References

[CR1] Adamsson A, Bernhardsson S (2018). Symptoms that may be stress-related and lead to exhaustion disorder: a retrospective medical chart review in Swedish primary care. BMC Fam Pract.

[CR2] Ahlborg G Jr, Hultberg A, Hadžibajramović E, Pettersson S, Ottosson E, Björk L, Lindegård Andersson A, Jonsdottir I (2016) *The KART-study. Work environment, stress and health among employees in the region of Västra Götaland.* ISM report nr 17. Retrieved from https://alfresco.vgregion.se/alfresco/service/vgr/storage/node/content/workspace/SpacesStore/e3676825-7ea8-4d07-919f-a9ec7e848add/2016_17_KART%20.pdf?a=false&guest=true Institutet för stressmedicin. Accessed 23 March, 2020

[CR3] Åkerlind I, Larsson R, Ljungblad C (2013). Leadership, social climate, health promotion, and sick absence – a comparable health care study in 60 municipalities (In Swedish: Ledarskap, socialt klimat, hälsofrämjande åtgärder och sjukfrånvaro – en jämförande studie inom vård och omsorg i 60 kommuner). Socialmedicinsk Tidskrift.

[CR4] Anderson TJ, Saman DM, Lipsky MS, Lutfiyya MN (2015) A cross-sectional study on health differences between rural and non-rural U.S. counties using the County Health Rankings. *BMC Health Serv Res* 15:441. doi:10.1186/s12913-015-1053-3 10.1186/s12913-015-1053-3 PMC4590732 26423746

[CR5] Befort CA, Nazir N, Perri MG (2012). Prevalence of obesity among adults from rural and urban areas of the United States: findings from NHANES (2005–2008). J Rural Health.

[CR6] Blumenberg C, Barros AJD (2018). Response rate differences between web and alternative data collection methods for public health research: a systematic review of the literature. Int J Public Health.

[CR7] Cohen J (1988). Statistical power analysis for the behavioral sciences.

[CR8] Cohen S, Kamarck T, Mermelstein R (1983) A global measure of perceived stress. J Health Soc Behav 24(4);385–396. Retrieved from https://www.ncbi.nlm.nih.gov/pubmed/6668417 6668417

[CR9] Ellbin S, Engen N, Jonsdottir IH, Nordlund AIK (2018). Assessment of cognitive function in patients with stress-related exhaustion using the Cognitive Assessment Battery (CAB). J Clin Exp Neuropsychol.

[CR10] Eskin M, Parr D (1996) *Introducing a Swedish version of an instrument measuring mental stress*. Reports from the Department of Psychology, no 813. University of Stockholm

[CR11] Gerber M, Jonsdottir IH, Arvidson E, Lindwall M, Lindegård A (2015). Promoting graded exercise as a part of multimodal treatment in patients diagnosed with stress-related exhaustion. J Clin Nurs.

[CR12] Glise K, Ahlborg G, Jonsdottir IH (2012). Course of mental symptoms in patients with stress-related exhaustion: does sex or age make a difference?. BMC Psychiatry.

[CR13] Glise K, Ahlborg G, Jonsdottir IH (2014). Prevalence and course of somatic symptoms in patients with stress-related exhaustion: does sex or age matter?. BMC Psychiatry.

[CR14] Glise K, Hadzibajramovic E, Jonsdottir IH, Ahlborg G (2010). Self-reported exhaustion: a possible indicator of reduced work ability and increased risk of sickness absence among human service workers. Int Arch Occup Environ Health.

[CR15] Grossi G, Perski A, Osika W, Savic I (2015). Stress-related exhaustion disorder – clinical manifestation of burnout? A review of assessment methods, sleep impairments, cognitive disturbances, and neuro-biological and physiological changes in clinical burnout. Scand J Psychol.

[CR16] Grossi G, Santell B (2009). Quasi-experimental evaluation of a stress management programme for female county and municipal employees on long-term sick leave due to work-related psychological complaints. J Rehabil Med.

[CR17] Hasselberg K, Jonsdottir IH, Ellbin S, Skagert K (2014). Self-reported stressors among patients with exhaustion disorder: an exploratory study of patient records. BMC Psychiatry.

[CR18] Health and Safety Executive HSE (2018) *Work related stress depression or anxiety statistics in Great Britain*, 2018. http://www.hse.gov.uk/statistics/causdis/stress.pdf. HSE. Accessed 23 March 2020

[CR19] Holmgren K, Dahlin-Ivanoff S, Björkelund C, Hensing G (2009). The prevalence of work-related stress, and its association with self-perceived health and sick-leave, in a population of employed Swedish women. BMC Public Health.

[CR20] Holmgren K, Fjällström-Lundgren M, Hensing G (2013). Early identification of work-related stress predicted sickness absence in employed women with musculoskeletal or mental disorders: A prospective, longitudinal study in a primary health care setting. Disabil Rehabil.

[CR21] Holtermann A, Krause N, van der Beek A, Straker L (2018). The physical activity paradox: six reasons why occupational physical activity (OPA) does not confer the cardiovascular health benefits that leisure time physical activity does. Br J Sports Med.

[CR22] Hultberg A, Ahlborg G Jr, Jonsdottir IH, Winroth J, Corin L (2018) *Health in the workplace, a complimation of knowledge and methods*. ISM-report nr 21. https://alfresco.vgregion.se/alfresco/service/vgr/storage/node/content/workspace/SpacesStore/1c23da66-62e1-4c47-bc34-bd8288aad0e6/2018%20_21_H%C3%A4lsa%20p%C3%A5%20arbetsplatsen.pdf?a=false&guest=true. Institutet för stressmedicin. Accessed 23 March 2020

[CR23] IBM (2017) IBM SPSS statistics for Windows. Version 25.0. Armonk, NY: IBM

[CR24] Jonsdottir IH, Nordlund A, Ellbin S, Ljung T, Glise K, Währborg P, Sjörs A, Wallin A (2017). Working memory and attention are still impaired after three years in patients with stress-related exhaustion. Scand J Psychol.

[CR25] Jonsdottir IH, Nordlund A, Ellbin S, Ljung T, Glise K, Währborg P, Wallin A (2013). Cognitive impairment in patients with stress-related exhaustion. Stress.

[CR26] Jonsdottir IH, Rödjer L, Hadzibajramovic E, Börjesson M, Ahlborg G (2010). A prospective study of leisure-time physical activity and mental health in Swedish health care workers and social insurance officers. Prev Med.

[CR27] Kallings L, Börjesson M (2014) *The best question about physical activity to patients*. http://www.gih.se/OM-GIH/Press-och-media/Pressmeddelanden/Basta-fragan-om-fysisk-aktivitet-till-patienter/ GIH. The Swedish School of Sport and Health Sciences. Accessed 23 March 2020

[CR28] Klingberg S, Mehlig K, Johansson I, Lindahl B, Winkvist A, Lissner L (2019). Occupational stress is associated with major long-term weight gain in a Swedish population-based cohort. Int Arch Occup Environ Health.

[CR29] Lee EH. Review of the psychometric evidence of the perceived stress scale (2012) *Asian Nurs Res* (Korean Soc Nurs Sci) (4):121–127. doi:10.1016/j.anr.2012.08.004 10.1016/j.anr.2012.08.004 25031113

[CR30] Lindroth M, Lundqvist R, Lilja M, Eliasson M (2014). Cardiovascular risk factors differ between rural and urban Sweden: the 2009 Northern Sweden MONICA cohort. BMC Public Health.

[CR31] McLaughlin D, Hockey R, Mishra G (2013). Heart disease in women in remote Australia: urban-rural differences after adjusting for lifestyle behaviours and socio-demographic factors. Aust N Z J Public Health.

[CR32] Mood C (2013). Life-style and self-rated global health in Sweden: a prospective analysis spanning three decades. Prev Med.

[CR33] National Board of Health and Welfare (2003) *Exhaustion disorder, stress-related disorders*. (In Swedish: Utmattningssyndrom, Stressrelaterad ohälsa). https://www.socialstyrelsen.se/globalassets/sharepoint-dokument/artikelkatalog/ovrigt/2003-123-18.pdf. Socialstyrelsen. Accessed 23 March 2020

[CR34] National Board of Health and Welfare (2020) *International statistical classification of diseases and related healh problems*. (In Swedish: Internationell statistisk klassifikation av sjukdomar och relaterade hälsoproblem). https://www.socialstyrelsen.se/globalassets/sharepoint-dokument/artikelkatalog/klassifikationer-och-koder/2020-2-6570.pdf. Socialstyrelsen,. Accessed 23 March 2020

[CR35] Nordin M, Nordin S (2013). Psychometric evaluation and normative data of the Swedish version of the 10-item perceived stress scale. Scand J Psychol.

[CR36] Norlund S, Reuterwall C, Hoog J, Lindahl B, Janlert U, Birgander LS (2010). Burnout, working conditions and gender – results from the northern Sweden MONICA Study. BMC Public Health.

[CR37] Oxenstierna G, Widmark M, Finnholm K, Elofsson S (2008a) *Psychosocial factors in today`s working life, and how to measure and describe them*. Stress Research Report, no. 320. Stress Research Institute, Stockholm

[CR38] Oxenstierna G, Widmark M, Finnholm K. Elofsson S (2008b) A new questionnaire and model for research into the impact of work and the work environment on employee health. *Scan J Work Environ Health Suppl*(6):150–162

[CR39] Pallant J (2016). *SPSS survival manual*: *A step by step guide to data analysis using IBM SPSS*.

[CR40] Persson R, Osterberg K, Viborg N, Jonsson P, Tenenbaum A (2016). The Lund University Checklist for Incipient Exhaustion – a cross-sectional comparison of a new instrument with similar contemporary tools. BMC Public Health.

[CR41] Peterson U, Bergstrom G, Demerouti E, Gustavsson P, Asberg M, Nygren A (2011). Burnout levels and self-rated health prospectively predict future long-term sickness absence: a study among female health professionals. J Occup Environ Med.

[CR42] Peterson U, Demerouti E, Bergstrom G, Samuelsson M, Asberg M, Nygren A (2008). Burnout and physical and mental health among Swedish healthcare workers. J Adv Nurs.

[CR43] Pietilainen O, Laaksonen M, Rahkonen O, Lahelma E (2011). Self-rated health as a predictor of disability retirement – the contribution of ill-health and working conditions. PLoS ONE.

[CR44] Salvagioni DAJ, Melanda FN, Mesas AE, Gonzalez AD, Gabani FL, Andrade SM (2017). Physical, psychological and occupational consequences of job burnout: a systematic review of prospective studies. PLoS ONE.

[CR45] SCB (2018) *Municipalites in numbers*. https://www.scb.se/hitta-statistik/sverige-i-siffror/kommuner-i-siffror/. SCB. Accessed 23 March 2020

[CR46] Schwartz R, Liu B, Sison C, Kerath SM, Breil T, Murphy L, Taioli E (2016). Study design and results of a population-based study on perceived stress following Hurricane Sandy. Disaster Med Public Health Prep.

[CR47] Sharon David H, Tenenbaum G (2017). The effectiveness of exercise interventions on coping with stress: research synthesis. Studies in Sport Humanities.

[CR48] Slavich GM (2016). Life stress and health: a review of conceptual issues and recent findings. Teach Psychol.

[CR49] Steene Johannessen J, Anderssen S, van der Ploeg HP, Hendriksen IJ, Donnelly AE, Brage S, Ekelund U (2016). Are self-report measures able to define individuals as physically active or inactive?. Med Sci Sports Exerc.

[CR50] Swedish Association of Local Authorities and Regions (2018) *Municipal employees 2018*. https://skr.se/ekonomijuridikstatistik/statistik/personalstatistik/personalenisiffror/tabellerkommunalpersonal2019/tabellerkommunalpersonal2018.32643.html. Sveriges kommuner och Regioner, Accessed 30 September 2020

[CR51] Swedish Association of Local Authorities and Regions (2019). *Sick absence in Municipalities and Regions*. (In Swedish: Sjukfrånvaro i kommuner och landsting). https://skr.se/tjanster/merfranskr/rapporterochskrifter/publikationer/sjukfranvaroikommunerochlandsting.27519.html. Sveriges Kommuner och Regioner. Accessed 30 September 2020

[CR52] Swedish Board of Agriculture (2015) *Our definition of rural areas* https://nya.jordbruksverket.se/stod/programmen-som-finansierar-stoden/var-definition-avlandsbygd.Jordbruksverket. Accessed 23 March 2020

[CR54] Swedish Social Insurance Agency (2020). *Social Insurance Report 2020:8. Mental disorder sick leave*. (In Swedish: Socialförsäkringsrapport 2020:8. Sjukfrånvaro i psykiatriska diagnoser.) Försäkringskassan. https://www.forsakringskassan.se/wps/wcm/connect/e12b777c-e98a-488d-998f-501e621f4714/socialforsakringsrapport-2020-8.pdf?MOD=AJPERES&CVID=. Accessed 30 September 2020

[CR55] Syssner J (2018) *Less many: about adaption and development in shrinking municipalities*. (In Swedish: Mindre många: om anpassning och utveckling i krympande kommuner). Dokument Press, Årsta

[CR56] Syssner L, Häggroth S, Ramberg U (2017) *To own the future: perspective on municipal development*. (In Swedish: Att äga framtiden: perspektiv på kommunal utveckling). LiU-tryck, Linköping

[CR57] Theorell T, Hasselhorn HM (2005). On cross-sectional questionnaire studies of relationships between psychosocial conditions at work and health–are they reliable?. Int Arch Occup Environ Health.

[CR58] Trivedi T, Liu J, Probst J, Merchant A, Jhones S, Martin AB (2015). Obesity and obesity-related behaviors among rural and urban adults in the USA. Rural Remote Health.

[CR59] Voss M, Stark S, Alfredsson L, Vingard E, Josephson M (2008). Comparisons of self-reported and register data on sickness absence among public employees in Sweden. Occup Environ Med.

[CR60] Wallensten J, Asberg M, Wiklander M, Nager A (2019). Role of rehabilitation in chronic stress-induced exhaustion disorder: a narrative review. J Rehabil Med.

[CR61] Wiegner L, Hange D, Bjorkelund C, Ahlborg G (2015). Prevalence of perceived stress and associations to symptoms of exhaustion, depression and anxiety in a working age population seeking primary care – an observational study. BMC Fam Pract.

[CR62] World Medical Association (WMA) (2013) *WMA declaration of Helsinki - ethical principles for medical research involving human subjects*. https://www.wma.net/policies-post/wma-declaration-of-helsinki-ethical-principles-for-medical-research-involving-human-subjects/. WMA. Accessed 23 March 2020

